# Pathophysiological Insights into a First-Onset Psychotic-Manic Episode Following Oseltamivir Use

**DOI:** 10.1192/j.eurpsy.2026.11765

**Published:** 2026-07-31

**Authors:** A. Uzturk, I. H. Karakus, S. S. Kirlioglu Balcioglu

**Affiliations:** Department of Psychiatry, Basaksehir Cam and Sakura City Hospital, Istanbul, Türkiye

## Abstract

**Introduction:**

Oseltamivir, a neuraminidase inhibitor for influenza A and B, is generally considered safe.It is converted by hepatic carboxylesterase 1 into oseltamivir carboxylate.Although trials support a favorable profile, post-marketing surveillance revealed rare neuropsychiatric complications requiring psychotropic treatment (Toovey *et al.*, 2008).Reported events, especially in children and young adults,include agitation,hallucinations,delusions,and mania (Ho *et al.*, 2010).Bipolar disorder itself arises from complex interactions between genetic vulnerability,neurotransmitter dysregulation and environmental triggers (American Psychiatric Association,2022).We describe a first-onset psychotic-manic episode following oseltamivir initiation,highlighting possible central nervous system (CNS) mechanisms.

**Objectives:**

To describe the clinical presentation,diagnostic work-up and management of a first-onset psychotic-manic episode after oseltamivir initiation,and discuss possible pathophysiological mechanisms.

**Methods:**

We present a single-patient case report. Investigations included urine toxicology, cranial MRI, EEG, complete blood count, biochemistry, coagulation studies,and viral serologies.Symptom severity was assessed with the Young Mania Rating Scale (YMRS) and Brief Psychiatric Rating Scale (BPRS).

**Results:**

An 18-year-old female with no psychiatric or family history developed reduced sleep, increased energy, pressured speech, grandiose religious delusions, and hallucinations on day five of oseltamivir (150 mg/day).Outpatient olanzapine was ineffective due to nonadherence, requiring hospitalization.Mental status examination confirmed a manic episode with psychotic features (DSM-5).Laboratory, neuroimaging, and EEG were normal.Lithium (1200 mg/day) and aripiprazole (up to 30 mg/day) produced substantial improvement within one week:YMRS decreased from 41 to 21 and BPRS from 52 to 31.She was discharged in stable condition.

**Conclusions:**

Although rare,neuropsychiatric complications of oseltamivir have been documented in clinical reports and pharmacovigilance data (Toovey *et al.*, 2008; Sakaeda *et al.*,2013).Their typical emergence within 2–7 days may relate to drug pharmacokinetics,hepatic conversion,and infection-induced blood–brain barrier (BBB) permeability (Yamashita *et al.*, 2009).Proinflammatory cytokines such as TNF-α and IL-6 may further compromise BBB integrity,facilitating CNS penetration.Preclinical evidence suggests oseltamivir carboxylate can modulate GABA-A receptor activity and dopamine transporter function, pathways implicated in mania (Chen *et al.*, 2019).In adolescents and young adults, reduced P-glycoprotein transporter function may enhance CNS drug accumulation,increasing vulnerability.This case illustrates a rare but clinically significant risk of oseltamivir-induced manic episodes.Vigilant psychiatric monitoring during antiviral therapy and timely intervention remain essential to patient safety.

**Disclosure of Interest:**

None Declared

